# Public appraisal of government efforts and participation intent in medico-ethical policymaking in Japan: a large scale national survey concerning brain death and organ transplant

**DOI:** 10.1186/1472-6939-6-1

**Published:** 2005-01-20

**Authors:** Hajime Sato, Akira Akabayashi, Ichiro Kai

**Affiliations:** 1 Department of Public Health, Graduate School of Medicine, The University of Tokyo, Hongo 7-3-1, Bunkyo-ku, Tokyo 113-0033, Japan; 2 Department of Biomedical Ethics, Graduate School of Medicine, The University of Tokyo, Hongo 7-3-1, Bunkyo-ku, Tokyo 113-0033, Japan; 3 Department of Social Gerontology, Graduate School of Medicine, The University of Tokyo, Hongo 7-3-1, Bunkyo-ku, Tokyo 113-0033, Japan

## Abstract

**Background:**

Public satisfaction with policy process influences the legitimacy and acceptance of policies, and conditions the future political process, especially when contending ethical value judgments are involved. On the other hand, public involvement is required if effective policy is to be developed and accepted.

**Methods:**

Using the data from a large-scale national opinion survey, this study evaluates public appraisal of past government efforts to legalize organ transplant from brain-dead bodies in Japan, and examines the public's intent to participate in future policy.

**Results:**

A relatively large percentage of people became aware of the issue when government actions were initiated, and many increasingly formed their own opinions on the policy in question. However, a significant number (43.3%) remained unaware of any legislative efforts, and only 26.3% of those who were aware provided positive appraisals of the policymaking process. Furthermore, a majority of respondents (61.8%) indicated unwillingness to participate in future policy discussions of bioethical issues. Multivariate analysis revealed the following factors are associated with positive appraisals of policy development: greater age; earlier opinion formation; and familiarity with donor cards. Factors associated with likelihood of future participation in policy discussion include younger age, earlier attention to the issue, and knowledge of past government efforts. Those unwilling to participate cited as their reasons that experts are more knowledgeable and that the issues are too complex.

**Conclusions:**

Results of an opinion survey in Japan were presented, and a set of factors statistically associated with them were discussed. Further efforts to improve policy making process on bioethical issues are desirable.

## Background

In Japan, it was not until 1997 that a law was finally enacted to legalize organ transplant from a brain-dead body. Since 1968, when the first heart transplantation from a person declared brain dead was performed, there have been long-standing struggles in Japan for and against this procedure. In addition to many non-governmental institutions and individuals, the Japanese government – both the legislature and administrative bodies – engaged in a variety of efforts for this enactment. A number of factors have been suggested for the prolonged lack of policy in this area: deep public mistrust of the medical profession caused by the 1968 heart transplant; the Japanese culture which still holds traditional Japanese view of death and the body; and the lack of the broad public consensus required as a precondition for a policy [1].

As elsewhere, public policy to resolve these social disputes was pursued [2]. Observers of the past policy process toward enactment offer contradictory evaluations: those for the speedy introduction of new medical technologies, for example, complain that possible organ recipients have suffered from the prolonged, impractical, and fruitless policy disputes, while those against hasty use of immature and controversial technologies have a good appraisal of the care exercised in past debates. Some argue that even now there are many unsettled issues. Previously, there has been no systematic study assessing past policy processes, factors that affect public appraisal of government efforts, and what future agenda items should include to ensure successful policy enactment.

In the policy process, which entails the introduction and implementation of a policy, public opinion is generally considered an important factor affecting its fate. Without favorable public opinion, a policy cannot be introduced and implemented effectively [3]. Public opinion regarding the process of policymaking is another important indicator of how well the government functions. This public appraisal essentially measures the degree of congruence between the public's expectations of government actions and perceived fulfillment of these ideals. When contending ethical value judgments are involved, satisfaction with the process affects the legitimacy of political institutions and processes. This, in turn, could influence the fate of proposed policies and condition future policy making [4]. Public opinion thus constitutes valuable information for determining how government bodies can and should proceed.

An important aspect in policy making is the degree of public participation, which is defined as a set of measures to consult with, involve, and inform the public to encourage participation in policy development [5]. Since experts, policymakers, and citizens all are limited in some aspects of their knowledge, public involvement is expected to improve the substantive quality of decisions, by incorporating public values, assumptions, and preferences. Public involvement also works toward educating the public, fostering trust in institutions, and reducing conflicts [6]. Citizen involvement legitimizes government efforts at policymaking, by lending credibility and thus increasing public trust in the political process.

This study examines public appraisal of past efforts of the Japanese government to legalize organ transplant from brain-dead bodies. Other goals are to quantify the public's intent to participate in future policy discussions and to identify possible factors affecting both appraisals and intentions to participate. A brief chronology of Japan's efforts since the 1960's toward legalization of organ transplant upon brain death is also presented. Implications of the study findings are discussed, as are suggestions for future research.

## Methods

### Questionnaire and subjects

A questionnaire includes sections on demographic characteristics (age, sex, education, occupation), health conditions (hospitalization in the past five years, current health condition), period of first issue attention (when did you first hear of the issue on brain death and organ transplant?), period of opinion formation (when did you arrive at the opinion you have now on this issue?), knowledge about donor card (are you familiar with the donor card, which indicates a personal directive to donate organs when determined to be brain-dead?), knowledge of past government efforts at informing the public and inviting their opinions (asking number of measures employed by the government), appraisal of past government efforts (How do you rate past government efforts around the issue of brain death and organ transplant?), and intention to participate in future (bioethics-related) policy discussions. Those who indicated unwillingness to participate were asked to provide reasons for that decision. The final questions concerned important agenda items for future policy processes.

Questionnaires were sent to 3000 people, selected from 15 cities and towns nationwide by the stratified random sampling method. They were requested to answer the questions on the sheets, and send them back by mail in a postage prepaid envelope. The study was conducted in January 2002, and the overall response rate was 34.5%.

### Statistical analysis

First, association of the questionnaire items both with a) appraisal of past government efforts and b) participation intent was examined using Mann-whitney tests (between two groups), Kruskal-Wallis tests (among multiple groups), and/or Spearman's rank correlations. Next, a multiple logistic regression model was applied to identify possible explanatory variables (entered and removed at the significance level of p = 0.05) to determine a set of variables that best predicts the dependent variables. Also, stepwise logistic regression analysis was conducted to determine factors affecting people's selection of important future agenda items.

### Additional materials

Extant opinion polls regarding public perceptions of brain death and organ transplants from brain-dead patients were studied to identify possible trends. When more than one poll was conducted in a year, results were averaged to avoid possible biases deriving from different survey designs. The following national polls were used to plot the trends in opinions: Yomiuri Shimbun (1982, 1984–95, 1997–99); Asahi Shumbun (1985, 1988, 1992, 1996–98); Mainichi Shimbun (1985, 1990–91, 1997); Office of the Prime Minister (1987, 1991, 1998); Nippon Hoso Kyokai (1991–92, 1996); and Jiji Tsushin (1992, 1994) [7].

## Results

Descriptive statistics indicating basic attributes of study participants, as well as all other survey results, are shown in Table 1. A relatively large percentage of people responded that they became aware of the issue either at the point when the Ministry of Health and Welfare (MHW) drew up the diagnostic criteria for brain death (23.7%), or when the Organ Transplant Act was adopted (33.6%). Most people (97.2%) were aware of the issue. The majority of respondents (69.9%) crystallized their opinions either when the Act was first adopted or around the time when the first organ transplant was conducted from a brain-dead donor. Close to half of the respondents (43.3%) were completely unaware of any governmental efforts regarding the issue, while the remainder were divided in their appraisal of those efforts (26.3% positive, 30.6% negative ratings). A majority (61.8%) responded that they would not participate in future policy discussions on bioethical issues.

**Table 1:** Description of subjects

Attribute	Category: number (frequency %); and/or average (sd)
Age	20s: 81 (8.1), 30s: 130 (13.1), 40s: 186 (18.7), 50s: 231 (23.2), 60s: 218 (21.9), over 70: 149 (14.9)
Sex	male: 491 (49.7), female: 498 (50.4)
Education	junior high: 170 (17.2), senior high: 459 (46.4), vocational: 87 (8.8), vocational high: 10 (1.0), community college: 72 (7.3), college: 182 (18.4), grad school: 10 (1.0)
Occupation	private enterprise: 268 (27.3), civil service: 58 (5.9), self-employed: 153 (15.6), part-time: 100 (10.2), housekeeping: 240 (24.4), student: 18 (1.8), others: 147 (14.9)

Hospitalization (past 5 years)	none: 756 (76.0), once: 181 (18.2), repeated for a disease: 33 (3.3), several times for multiple reasons: 25 (2.5)
CurrentHealthCondition	healthy: 359 (36.2), relatively healthy: 545 (55.0), unhealthy: 87 (8.8)

FirstAttentionPeriod	Per1 : 230 (23.7), Per2: 114 (11.8), Per3: 138 (14.2), Per4: 326 (33.6), Per5: 135 (13.9), Per7: 27 (2.8)
OpinionFormationPeriod	Per2: 97 (11.0), Per3: 73 (8.3), Per4: 257 (29.2), Per5: 188 (21.4), Per7: 265 (30.1)

KnowDonorCard	yes: 894 (91.0), no: 89 (9.1)
KnowGovtEfforts (score range: 0–7)	0: 92 (9.2), 1: 295 (29.5), 2: 233 (23.3), 3: 168 (16.8), 4: 129 (12.9), 5: 60 (6.0), 6:22 (2.2); average: 2.22 (1.48); Cronbach's alpha: 0.603

Appraisal of past governmental efforts (score range: 1–5)	sufficient: 35 (3.6), relatively sufficient: 222 (22.7), relatively insufficient: 178 (18.2), insufficient: 121 (12.4), do not know any efforts: 424 (43.3) Average:3.69 (1.32)
Participation intent(score range: 1–4)	yes: 35 (3.5), relatively yes: 344 (34.7), relatively no: 531 (53.5), no: 82 (8.3)

Note: Notation of Periods; Per1:1985 (MHW Brain death standard), Per2:1989 (Brain death ad hoc council set), Per3:1992 (Brain death ad hoc council rep), Per4:1997 (Law adopted), Per5:1999 (TPBD first conducted), Per6:2002 (15 cases done), Per7:(not yet accepted, not yet decided, not interested).

KnowGovtEfforts: Score of the knowledge about the past government efforts, calculated as the number of items known. Items comprise Opinion polls & hearings, Expert councils, Public statements and announcements, Referendum, Town meeting & roundtables, Public comments, and Others.

**Figure 1:**
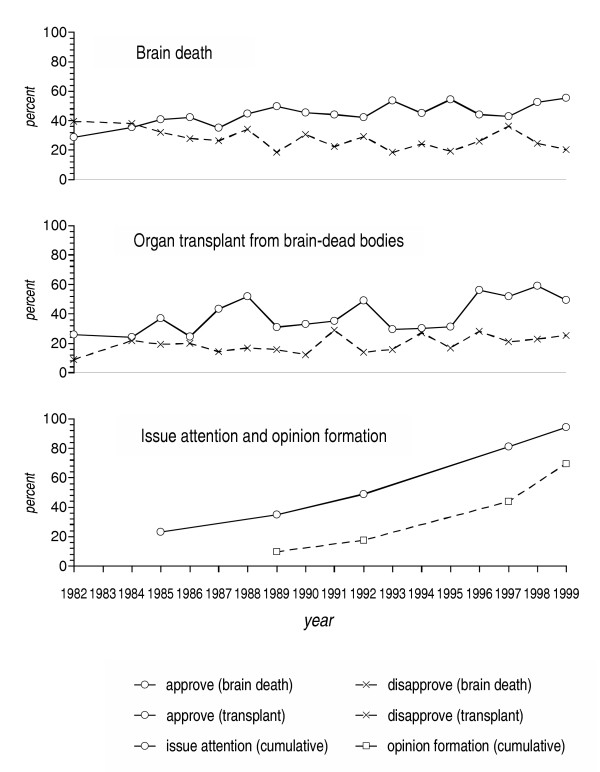
Trend of public opinion on brain death and organ transplant in Japan

Figure 1 shows the general trends in public opinion concerning brain death and organ transplant from brain-dead bodies, the cumulative proportion of people who attended to the issue, and the cumulative proportion of people who initially formed their personal opinions at the point of study. Since the early 1980, Japanese acceptance of the concept of brain death steadily increased, while disapproval minimally declined. As public acceptance of organ transplants from brain-dead bodies increased, so did public opposition, meaning that more people were forming opinions than in the past. This is further indicated in the trend study: The proportion of people attending to the issue consistently increased over time, as did solidification of personal opinions, although on a smaller scale.

Table 2 presents the correlation of factors associated with appraisal of past government efforts or personal intent to participate in future policy discussion. Sex, time period of first attention to the issue, period of opinion formation, knowledge of donor card, and knowledge of past government efforts were significantly associated with appraisal. On the other hand, age, education, period of first attention to the issue, period of opinion formation, and knowledge of past government efforts were associated with participation intent. For both appraisal and participation, there were similar tendencies observed. Respondents indicate more positive appraisals or greater participation intent when they are older, male, attended to or formed opinion of the issue earlier, are aware of the donor card, and more knowledgeable about past governmental efforts.

**Table 2:** Determinants of public appraisal towards governmental efforts, and of public intention to participate in policy discussion (Bivariate analysis)

	Appraisal score [best = 1, worst = 5]		Participation score [most = 1, least = 4]	
	Category: average appraisal score + sd, and/or Spearman's correlation coefficient (rho)	p-value	Category: average appraisal score + sd, and/or Spearman's correlation coefficient (rho)	p-value

Age	rho = -0.062	0.053 (3)	rho = -0.085	0.009(3)
	male (3.58 + 0.06), female (3.80 + 0.06)	0.002 (1)	male (2.64 + 0.71), female (2.71 + 0.03)	0.086 (1)
Education	junior high (3.73 + 1.39), senior high (3.76 + 1.37), vocational (3.1 + 1.20), vocational high (3.69 + 1.31), community college (3.73 + 1.28), college (3.83 + 1.47), grad school (3.17 + 1.47)	0.545 (2)	junior high (2.73 + 0.75), senior high (2.73 + 0.61), vocational (2.60 + 0.67), vocational high (2.70 + 0.48), community college (2.69 + 0.75), college (2.49 + 0.73), grad school (2.36 + 0.67)	0.004 (2)
Occupation	1 (3.74 + 1.35), 2 (3.80 + 1.27),3(3.79 + 1.36), 4 (3.92 + 1.30), 5 (3.79 + 1.41), 6 (3.42 + 1.30), 7 (3.60 + 1.38)	0.746 (2)	1(2.63 + 0.66), 2(2.60 + 0.72),3(2.64 + 0.67), 4(2.70 + 0.69), 5(2.70 + 0.66), 6(2.32 + 0.75), 7(2.71 + 0.69)	0.287 (2)

Hospitalization 5 yrs	rho = -0.021	0.510 (3)	rho = 0.009 0.614 (2)	0.777 (3)
CurrentHealthCondition	rho = 0.013	0.689 (3)	rho = 0.042 0.343 (2)	0.200(3)

FirstAttentionPeriod	Per1 (3.52 + 1.37), Per2 (3.75 + 1.33), Per3 (3.50 + 1.34), Per4 (3.73 + 1.37), Per5 (4.30 + 1.18), Per7 (4.31 + 1.46); rho = 0.210	0.000 (2) 0.000 (3)	Per1 (3.52 + 1.37), Per2 (3.75 + 1.33), Per3 (3.50 + 1.34), Per4 (3.73 + 1.37), Per5 (4.30 + 1.18), Per7 (4.31 + 1.46); rho = 0.181	0.000(2) 0.000(3)
OpinionFormationPeriod	Per2 (3.40 + 1.30), Per3 (3.32 + 1.39), Per4 (3.45 + 1.34), Per5 (3.68 + 1.32), Per7 (4.27 + 1.24), others(3.98 + 1.38); rho = 0.269	0.000 (2) 0.000 (3)	Per2 (3.40 + 1.30), Per3 (3.32 + 1.39), Per4 (3.45 + 1.34), Per5 (3.68 + 1.32), Per7 (4.27 + 1.24); rho = 0.204	0.000(2) 0.000 (3)

KnowDonorCard	yes (3.62 + 1.32), no (4.31 + 1.23)	0.000 (1)	yes (2.66 + 0.67), no(2.80 + 0.70)	0.084(1)
KnowGovtEfforts	rho = -0.083	0.009 (2)	rho = -0.175	0.000(3)

Participation intent	rho = 0.061	0.056 (3)		
Appraisal score			rho = 0.061	0.056(3)

Note: Significance in appraisal score across groups were tested by (1) Mann-whitney tests (between two groups), (2) Kruskal-Wallis tests (among multiple groups), and/or (3) Spearman's rank correlations.

Moreover, when people are older, they are more likely to have recognized the issue and formed their opinions earlier, but know less about donor cards and past governmental efforts. Knowledge of donor cards and of government efforts were positively correlated, as were period of first attention to the issue and period of opinion formation. There was no significant correlation between appraisal and participation intent.

Table 3 shows the results of multiple regression analyses, with the appraisal score and the participation intent score as dependent variables. A combination of age, period of opinion formation, and knowledge of the donor card best predicts public appraisal of the past governmental efforts, while a combination of age, period of first attention to the issue, period of opinion formation, and knowledge of governmental efforts best predicts individual intention to participate in future policy discussions. Individuals had more favorable appraisals of government actions when they were older, formed their opinions earlier, and knew about donor cards. On the other hand, individuals indicated greater intention to participate in future policy discussions when they were younger, paid attention to the issue earlier, formed their opinions earlier, and had more knowledge about past government efforts.

**Table 3:** Predictors of appraisal/participation (stepwise multiple regression analysis)

	Selected independent variables (coeff + sd, p-value, 95% CI)	Model adjusted-R2 (p-value)
Appraisal score as a dependent variable [best = 1, worst = 5]	Age (-0.059 + 0.030, 0.049, -0.117 - -0.000)Opinion Formation Period (0.196 + 0.027, 0.000, 0.144 - 0.248) Know Donor Card (0.583 + 0.168, 0.001, 0.253 - 0.913)	0.085 (0.000)

Participation intent as a dependent variable [most = 1, least = 4]	Age (0.043 + 0.016, 0.008, 0.011 - 0.075) First Attention Period (0.053 + 0.017, 0.002, 0.019 - 0.087) Opinion Formation Period (0.064 + 0.015, 0.000, 0.034 - 0.093) Know Govt Efforts (-0.048 + 0.016, 0.003, -0.080 - -0.017)	0.070 (0.000)

Note: Age (20s = 1, 30s = 2, 40s = 3, 50s = 4, 60s = 5, over70 = 6), Know Donor Card (know = 0, do not know = 1), Notation of other variables explained in Table 1 (Note).

As shown in Table 4, respondents (42.3–69.7%) indicated that the opinions of patients, experts, and citizens should be more respected in policymaking, and also that more information disclosure is desirable. About 20% of people thought that the time spent for policymaking was not appropriate, either too long or too short. Very few people believed that government opinion should be weighted more, or that the process timeline is just right (status quo). Bivariate analyses disclosed no significant relationship of the selection of particular future agenda items either with the appraisal of government efforts or with participation intent. Results of multiple logistic regression analysis indicated that knowledge of past government efforts is related to all the future agenda items, except for one (more government opinion). Among those items associated, all but status quo were more likely to be chosen as knowledge of past government efforts increased.

Those who were unwilling to participate in future policy discussion cited several reasons for their decision: Experts know better (50.1%); Issue is difficult (44.9%); Participation is ineffective (21.6%); Too busy (14.1%); and Not interested (7.9%).

**Table 4:** Important future agenda and their predictors

Agenda	Yes/No: numbers (%)	Predictors of respondents who selected the items: selected independent variables (odds ratio + sd, p-value, 95% CI)	Model peudo-R2 (p-value)
More disclosure	Yes: 695 (69.6) No: 304 (30.4)	Education (1.178 + 0.060, 0.001, 1.065 - 1.302) KnowGovtEfforts (1.629 + 0.112, 0.000, 1.424 - 1.865) FirstAttentionPeriod (0.873 + 0.049, 0.016, 0.782 - 0.975)	0.104 (0.000)
More citizen opinion	Yes: 423 (42.3) No: 576 (57.7)	KnowGovtEfforts (1.267 + 0.061, 0.000, 1.153 - 1.393)	0.021 (0.000)
More patients' opinion	Yes: 696 (69.7) No: 303 (30.3)	Age (0.853 + 0.048, 0.005, 0.764 - 0.952) Sex (1.593 + 0.257, 0.004, 1.161 - 2.186) KnowGovtEfforts (1.443 + 0.088, 0.000, 1.280 - 1.627)	0.064 (0.000)
More experts' opinion	Yes: 565 (56.6) No: 434 (43.4)	Age (1.122 + 0.056, 0.020, 1.018 - 1.236) KnowGovtEfforts (1.392 + 0.073, 0.000, 1.255 - 1.543)	0.038 (0.000)
More gov't opinion	Yes: 9 (0.9) No: 990 (99.1)	Age (2.337 + 0.836, 0.018, 1.160 - 4.712)	0.098 (0.006)
More time	Yes: 211 (21.1) No: 788 (78.9)	Age (1.214 + 0.077, 0.002, 1.071 - 1.375) Health condition (1.686 + 0.247, 0.000, 1.265 - 2.246) KnowGovtEfforts (1.255 + 0.073, 0.000, 1.119 - 1.407)	0.043 (0.000)
More Speedy process	Yes: 205 (20.5) No: 794 (79.5)	Age (0.880 + 0.052, 0.030, 0.784 - 0.988) OpinionFormationPeriod (0.875 + 0.046, 0.011, 0.789 - 0.969) KnowGovtEfforts (1.243 + 0.071, 0.000, 1.112 - 1.391)	0.034 (0.000)
Status quo	Yes: 12 (1.2) No: 987 (98.8)	KnowGovtEfforts (0.182 + 0.093, 0.001, 0.067 - 0.494)	0.199 (0.000)

Possible explanatory (independent) variables for stepwise logistic regression analysis: KnowGovtEfforts, Age, Sex, Education, Occupation, Hospitalization, CurrentHealthCondition, FirstAttentionPeriod, OpinionFormationPeriod, KnowDonorCard, Appraisal score, and Participation intent.

## Discussion

Using data obtained in an opinion survey, this study seeks to evaluate public appraisal of past government efforts to legalize organ transplant from brain-dead bodies in Japan. Even though public opinion is relatively unstable and sometimes an irrational response to surrounding symbols, it continues to be an important factor in policy advocacy. Public acceptance of a policy is considered important not only for the practical reason that adopted policy cannot be implemented effectively and efficiently without public consent, but also for the ideological democratic point of view that policy is to be based on the judgment of a rational, informed and willing public, or at least on conscious delegation of individual autonomy to expert authorities [8]. The degree of public approval also conditions the future course of events. In this context, efforts to keep relevant public informed are quite important, as are those toward the accomplishment of public policymaking. Policy advocates should generate and meet public expectations of responsible and reliable government actions, either by assuming a leadership role to generate public expectations or by taking a conforming posture to help meet those expectations.

### Chronology of the act on organ transplant in Japan

The chronology of the major events leading to enactment of the Organ Transplant Act and the successful implementation of the first several operations in Japan has been described elsewhere in detail [9]. Here we present a succinct summary.

An act which enabled cornea transplant operations with the consent of the family was passed in 1957. In the same time period, kidney (1956-) and liver (1964-) transplants also commenced. The first heart transplant in Japan was performed at Sapporo Medical College in 1968. Since brain death had not been officially established, there was concern about a possible conflict of interest, and some believed that the extraction of a heart was murder. Extended efforts have taken place since then, to develop criteria for death, specifically medical and biological definition and diagnosis, their social use, and how to determine a human/ individual death in relation to the criteria.

In the medical field, a committee of the EEG Society published standards for diagnosing brain death in 1974. The donor card system was sanctioned by the government shortly thereafter in 1977, without defining the criteria for death. In the meantime, organ transplant was sporadically performed with the consent of families, but without any official regulation of the process. Consequently, the transplant of cadaveric organs spread only gradually, since harvesting organs from brain-dead bodies continued to invoke public dispute and sometimes resulted in lawsuits. In 1984, for example, when the first multiple transplant (a combined kidney/pancreas transplant) was performed from a brain-dead body at Tsukuba University, the Patients' Rights Conference (PRC) soon filed a charge of murder in the case.

The medical community began to advocate legislation governing brain death and organ transplantation more openly. In 1985, the MHW announced its diagnostic criteria for brain death, though it stated that a patient's death couldn't be judged by brain death. [Period 1]. In 1986, the Japan Medical Association formed the Bioethics Discussion Group, a study group of interdisciplinary nature, and in 1988 issued its Final Report, which encouraged brain death legislation to facilitate organ transplantation. With the goal of shaping public opinion, the Japan Organ Transplantation Society sponsored a series of open symposia in 1989. [Period 2]. A group of politicians from the major party started investigating the current situation in other countries, considering possible legislation. Activities of patients' groups reportedly helped shift public attention away from the brain-dead potential donor to the seriously ill person who needs a transplant [10,11]. At the same time, opposition was increasing, especially from the PRC, the Japanese Society of Psychiatry and Neurology, and the Japan Federation of Bar Associations. These groups called for a (social) consensus, unitary and conclusive definition of death [12]. Due to these public debates and advocacy efforts, media coverage on brain death and organ transplant increased dramatically.

Finally, in early 1990, more than 30 years after the first transplant, the office of the Prime Minister established a special commission, Provisional Commission for the Study of Brain Death and Organ Transplantation. To encourage public involvement, the Commission held a series of public hearings and town meetings, and issued newsletters. Its 1991 interim and 1992 final reports presented both a majority view and a minority view. The former stated that a social consensus on brain death had already been achieved, and the latter argued that such a consensus had not yet been achieved. Both groups approved organ transplant when the consent of the donor was definitely obtained. A number of scholars argued that it should be a personal decision whether or not one's death is to be determined by brain death criteria, making individual consent the basis of both brain death and organ donation [13]. [Period 3].

A bill to legalize the transplantation of organs was presented to the Diet first in 1994. After several years of discussion, the modified bill finally became law in October 1997. Organ transplantation was thereby legalized where the donor has given written consent both to transplant and to the determination of brain death. Brain death was accepted only in such a case to enable organ transplants. In practice, the patient's family can still override the prior consent decision. [Period 4]. In 1999, two years after the law was passed, the first heart transplantation was successfully conducted, at Osaka University Hospital [14]. That same year, the second and third cases were successfully operated at the National Cardiovascular Center. [Period 5]. Though several issues, such as privacy protection and information disclosure, coordination of donors and recipients, as well as medical expense coverage, were raised during this series of successful operations, strong opposition to the law was no longer voiced. By 2002, 15 organ transplants had been conducted from brain-dead bodies. [Period 6].

### Issue attention and opinion formation

As was indicated in Figure 1, though it is difficult to make qualitative judgments, the extended debates and struggles helped increase public awareness and knowledge of the issue, leading many to form policy preferences. More than anything else, the official diagnostic guidelines for brain death (1985) and the legislation (1997) increased public attention to the issue, and facilitated opinion formation (Proportion of people varied across time periods significantly at p = 0.05, by chi-squared tests). These official actions, accompanied by wide media coverage, mobilized a previously inattentive public through their social conspicuousness.

Our results also indicate that as the number of people approving organ transplants from brain-dead bodies increased, the number of opponents increased in parallel, although in smaller numbers. This increase in political awareness or in political knowledge, as suggested elsewhere [15], led to increased polarization of attitude reports, resulting in the wider division between policy opinions. It should also be noted, however, that significant numbers of people had not formed their opinions until the first case of organ transplant was (successfully) achieved, and that about 30% of people remain undecided. The former appear to have waited to see what were the real consequences of the technology, i.e., its success or failure, as well as the social reaction. The latter are either watching for future developments or uninterested.

As in other countries, the public debates on brain death and organ transplant, both in the private sphere and in the public sphere, were new attempts at governance of socio-technical innovation in the field of biomedicine. If social mobilization is fueled by the inability of the institutional system to respond adequately to public concerns, the issue status in Japan in the 1980s, when many individuals and institutions started to pay attention and get involved in the debate, might indicate insufficient mediation of the actors for conflict resolution (by the government) before and during that period. Generally in post-war Japan, a relatively small number of political and administrative elites have left the handling of many social conflicts to the workings of traditional social relations [16]. Similarly, the government, for a long time, largely left issue of brain death to medical communities and to a set of mobilized individuals and groups. The sporadic but recurrent implementation of transplant operations under no official rules made latent value conflicts manifest, randomly shaping the political landscape. Although lack of government action exacerbated social disputes, which in turn inhibited government intervention, it helped increase public awareness of the issue. Official actions were thus preceded by these social disputes.

In the early 1990s when the Ad-hoc Council was set up, the Japanese government introduced a variety of measures to resolve social disputes by inviting the public into policy discussions. Our finding that many people recognized the issue and formed their opinions at times other than this period, however, suggests that these tactics were not very effective in terms of raising public awareness. In European countries, it was reported, public involvement measures served well as focusing devices, which helped attract attention and facilitate discussion among the various public [17]. The difference between Japan and European countries might be attributed to the difference in their participatory nature, as expected and instilled by these measures. At face value, the Japanese measures are designed to increase public involvement and active mobilization, but instead they function more as mechanisms for public consultation. A widespread norm of situational decision making, perceiving events and making judgments while experiencing them, could also induce people to reserve their judgments and opinion formation until implementation [18]

### Public appraisal of the past government efforts

It is remarkable that more than 40% of respondents were unaware of any past government policy, despite longstanding struggles around the issue and much media coverage. Of those who were aware, about half of them had favorable opinions of government efforts, while a slightly larger percentage had negative views. The fact that only 30% of respondents reported satisfaction indicates that there is much room to improve public awareness, acceptance, and appraisal in the policy process on bioethical issues.

Multiple regression analysis disclosed that age, period of opinion formation, and knowledge of donor cards are independent factors affecting public appraisal of past governmental efforts. Individuals tended to give higher appraisal points when they were older. Indeed, age seems to be a major factor in opinion formation on several policy issues [19]. Perhaps older people are more concerned with the issue of death and how it is defined because of its imminence and also because older people are more inclined to the traditional and community-oriented viewpoints. This in turn makes them more aware of and inclined to accept and praise a careful policy deliberation process. Younger people are, on the other hand, more free from traditional values and more concerned about individual rights and liberty, as was suggested by our finding that younger people were more likely to indicate that greater respect for patients' opinions and a speedier process are important items for future agendas. They could therefore have been frustrated by the time-consuming search for a social and unified definition of death. The finding that a higher appraisal was given by those people who made up their minds by the early debates and events, and by those who were aware of the donor card, might suggest that these people again value the freewheeling but deliberate process without any dictatorship.

A national effort to incorporate ethical considerations into policy rests on an academic reservoir of technical experts, legal scholars and humanists, and on the public understanding of science and its social implications, as well [20]. The prolonged absence of direct leadership or clear policy provided society with ample time and opportunity for public debate, which is a collective learning process through a set of exchanges of viewpoints and/or social confrontations. After the early struggles which searched for unified value judgments, the policy discussions gradually shifted more to the social rules allowing adversarial opinions. Through these deliberations, many people have come to realize that organ transplant can be an acceptable and promising medical therapy so long as the donor's human rights are protected, and that the policy can protect the common good by tolerating divergent values and allowing individual choice of death criteria at the time of organ donation. This long social debate, which bore fruit in the enactment of a law, was considered an acceptable and even necessary step, by attentive members of the public, even though the debates were not necessarily strategically planned.

According to Taylor and Fiske [21], people react critically to the arguments they encounter only to the extent that they are knowledgeable about political affairs. Hill [22] argues that ordinary citizens are rational only to a limited extent, but capable of good judgment when they have access to reliable facts and interpretations prepared by experts. In light of these arguments, the finding that the more attentive members of the public provided a better appraisal is promising, especially if those appraisals are more informed and rational than those rendered by the less attentive. If measures are taken to better inform the public of past policy discussions and of current policy as well, the public could be more content with government activities. In this context, the expert role of informing the public of past policy discussions would be critical to nurturing opinions [23].

In relation to this point, it should be noted that some changes in the policymaking process were regarded as important. A majority of respondents suggested that better information disclosure and more respect for both patients' and experts' opinions are desirable in the policy process. It follows that more effective involvement of the public, especially those stakeholders, in policymaking is warranted. Although an empirical assessment is not available, some procedures used in France and Germany might merit attention in modeling future policymaking for other countries. The National Consultative Bioethics Committee of France holds an annual public conference where, in addition to an activity report from the Committee, many ethical topics are discussed, with the participation of both experts and laypeople [24]. The German Reference Center for Ethics in the Life Sciences functions as a clearinghouse and library, open both to researchers, policymakers, and the public, while the German National Ethics Council holds conferences and issues newsletters both of which are open to the public [25]. Regular activities of this kind, targeting and involving the public, could be expected to increase the base of attentive, informed, and rational citizenry.

### Public involvement in the future

In many countries, participation has gained momentum in a variety of policy domains [26]. In health care decisions, public participation, public involvement, or public consultation is considered desirable and even necessary by both policymakers and members of the public [27]. The participation process is used to obtain information from, and to provide information to, the community, which helps ensure fair, transparent and legitimate decision-making and garner support for the outcomes of the process [28,29].

In our study, a majority of people (61.8%) responded that they would not participate in future policy discussions, while the rest (38.2%) responded that they would. The absence of association between government appraisal and participation intent indicates that the latter is determined by factors other than the former. Multivariate analysis showed that younger age, earlier period of first attention, earlier period of opinion formation, and more knowledge of past governmental efforts are positively associated with the intent to participate in future policy discussions. This indicates that the more attentive members of the public, namely those long-term observers with their own opinions and knowledge of current policy, have more interest and consequently a greater intention to participate in policy discussions. This finding is consistent with past studies indicating that participation is facilitated by policy knowledge and/or political sophistication [30]. Positive association of knowledge of past governmental efforts with participation intent might, more specifically, suggest that a variety of measures newly employed by the government to incorporate public opinion, such as public hearings and comments, town meetings, and expert councils, were welcomed by the public, and somehow inspired their participation in the policymaking process.

Reasons cited for being unwilling to participate in the process indicate that many feel unqualified or unknowledgeable but not necessarily too busy or uninterested. Further analysis revealed that older people are more likely to cite "Experts better" and "Ineffective", and are less likely to choose "Busy"; that females are more likely to cite "Difficult", and that people tend to cite "Ineffective" when they are more knowledgeable about past government efforts, while choose "Busy" or "Uninterested" when they are less knowledgeable. These findings suggest that despite their latent interest in the issue, people are unwilling to commit themselves to policy discussions because of their perception of inadequacy stemming both from their lack of knowledge and sense of inefficacy, as judged from past experiences. The absence of an association between appraisal and participation suggests that people might be uncertain about their own competence and efficacy in policymaking. It can be inferred that people hope for a means of understanding the issue, so as to formulate their opinions for themselves.

Political participation is facilitated by having a personal stake and perceived self-efficacy in policymaking. Conversely, it could be hampered by both indifference to the issue and a sense of powerlessness [31]. More specifically, the factors influencing participation encompass the structural and social context of the population as predisposing factors (e.g., income and education), the institutional context for decision-making as an enabling factor (e.g., the activities of media, governments and other institutions), and the role of interests and interest groups as precipitating factors. Different degrees of public participation and different kinds of public involvement measures could be potent enabling factors affecting participation intent [32]. People are more willing to be involved in decision-making when there is a guarantee that their contributions will be heard and that decisions taken following consultation will be explained [33]. For the public to be effectively mobilized into policy debates, they must feel assured they are sufficiently informed and can assume an important role in policymaking [34]. Essentially, people will become involved if they believe they have the proper tools and their efforts will make a difference.

In the context of Japan, it is important to remember that public involvement measures thus far used were not very effective in raising public awareness and that people consider some changes desirable in the policymaking process. It is possible that more people could be inspired to participate by changing the design of public involvement measures, from a consultation type of involvement to a partnership model [35]. Efforts to keep people informed, to help them understand the issues, to generate spheres for public deliberation, while at the same time creating mechanisms to ensure their voices will be heard in the policy process, could help mobilize the public and facilitate a discursive formation of opinion among them [36]. Both the public and the policymakers should acknowledge the important role citizens can play in policy discussions around biomedical ethics [37].

More innovative methods of public participation show promise and deserve consideration in improving policy process on medico-ethical issues and increasing public satisfaction with policy and politics. These include consensus conferences, citizens' juries, scenario workshops, deliberative opinion polls, among others [38]. Distinct from traditional opinion surveys, these methodologies seem intended to redress the deficiencies in citizen ability, such as limited expertise and attention to the issue among laypeople, and seek to elicit informed and rational choices. In some cases, debates at conferences are publicized through mass media, to raise public awareness of the issue and invite further social discussion [39,40]. Though overall satisfaction with and acceptance of these measures by the public has not been fully documented, the innovative methods reportedly had considerable success in increasing public awareness of an issue and facilitating logical and comprehensive discussion, which served as the basis for subsequent legislation [41].

### Study limitations and future research agenda

As is always the case with mass opinion surveys, this study cannot escape the possible bias introduced by the low response rates of polls [42]. A sampling with a disproportionately large number of the attentive public, omitting those with moderate positions, may result in opinion polarization, exaggerating the true conditions, while missing attentive part of the public can cause opinion neutralization, overlooking some important traits. These issues should be addressed in future research, hopefully also validating findings through the comparison of different studies. As was noted above, public participation in policy-making is a trend in many countries. The generalizability of our findings should be carefully tested by empirical studies.

Among many topics to be considered for future research is the function of (mass) media vis-à-vis public opinion formation. The media should be examined critically as they influence both experts, policymakers, and the public. Mass media have a dual function in these processes: as a conduit of debates and negotiations as well as a source of influence [43]. Also on public side, the possibility of an active role for audiences in meaning creation should be explored. This study, without directly asking individual policy preferences, fell short of proposing or validating any theoretical model of opinion formation. The accumulation of knowledge by experimental and innovative public participation measures, such as deliberative polls, might answer some of these as yet unanswered questions.

## Conclusions

Government decisions and their outcomes, namely the enactment and subsequent implementation of organ transplants, attracted public attention and helped formulate public opinion on the issue, more than did the processes leading to enactment. In the case of the concept of brain death and the legalization of organ transplant in Japan, many people still were unaware of past government efforts in policymaking, including the measures used for public involvement, despite past longstanding social debates. Only a small percentage of the public indicated satisfaction with the process. However, those who were attentive to the issue, knowledgeable of the past policy process as well as of the current policy, tended to rate the policy process more positively. Although people do not always manifest their intent to participate in future policy discussions, they might maintain sufficient interest in biomedical issues and have a latent wish to get involved in the policy process.

## Competing interests

The author(s) declare that they have no competing interests.

## Authors' contributions

All the authors (HS, AA, IK) fully participated in the planning, designing, and carrying out of the surveys for this study. HS performed the statistical analysis and drafted the article. All authors read and approved the final manuscript.

## Pre-publication history

The pre-publication history for this paper can be accessed here:



## References

[B1] Feldman EA (1994). Culture, conflict, and cost: Perspectives on brain death in Japan. International Journal of Technology Assessment in Health Care.

[B2] Sass HM, ten Have HAMJ, Sass HM (1998). Action driven consensus formation. Consensus formation in healthcare ethics.

[B3] Jones CO (1978). An Introduction to the Study of Public Policy.

[B4] Manin B (1987). On legitimacy and political deliberation. Political Theory.

[B5] Rowe G, Frewer LJ (2000). Public participation methods: A framework for evaluation. Science, Technology and Human Values.

[B6] Leroux T, Hirtle M, Fortin LN (1998). An overview of public consultation mechanisms developed to address the ethical and social issues raised by biotechnology. Journal of Consumer Policy.

[B7] Akabayashi A (2003). Report of the project "Strategies for Social Consensus Building on the Policies concerning Advanced Medical Technologies.

[B8] King CS, Feltey KM, Susel BO (1998). The question of participation: Toward authentic public participation in public administration. Public Administration Review.

[B9] Brannigan MC (1995). A chronicle of organ transplant progress in Japan. Transplant International.

[B10] Kimura R (1991). Japan's dilemma with the definition of death. Kennedy Institute of Ethics Journal.

[B11] Kita Y, Aranami Y, Nomura Y, Johnson K, Wakabayashi T, Fukunishi I (2000). Improving public awareness of organ transplantation in Japan. Progress in Transplantation.

[B12] Hardacre H (1994). Response of buddhism and shinto to the issue of brain death and organ transplant. Cambridge Quarterly of Healthcare Ethics.

[B13] Ad-hoc Committee on Brain Death and Organ Transplantation (1991). Interim Report (Important issues related to Brain Death and Organ Transplantation).

[B14] Hori M, Yamamoto K, Kodama K, Takashima S, Sato H, Koretsune Y, Kuzuya T (2000). Successful launch of a cardiac transplantation in Japan. Japanese Circulation Journal.

[B15] Zaller JR (1992). The nature and origins of mass opinion.

[B16] Pharr SJ (2000). Losing face: Status politics in Japan.

[B17] Joly PB, Assouline G (2001). Assessing Public Debates and Participation in Technology Assessment in Europe: ADAPTA Project Final Report.

[B18] Akabayashi A, Slingby BT, Kai I (2003). Perspectives on advance directives in Japanese society: A population-based questionnaire survey. BMC Medical Ethics.

[B19] Tourangeau R, Rasinski K, Bradburn N, D'Andrade R (1989). Carryover effects in attitude surveys. Public Opinion Quarterly.

[B20] Cook-Deegan RM, ten Have HAMJ, Sass HM (1998). Finding a voice for bioethics in public policy: Federal initiatives in the United States, 1974–1991. Consensus formation in healthcare ethics.

[B21] Taylor SE, Fiske S, Berkowitz L (1978). Salience, attention, and attribution: Top of the head phenomena. Advances in social psychology.

[B22] Hill S (1992). Democratic values and technocratic choices.

[B23] Brunner RD, Lasswell HD, Lipset SM (1995). The Encyclopedia of democracy.

[B24] CCNE (Comite Consultatif National d'Ethique) (2002). Les Chahiers du Comite Consultatif National d'Ethique pour les Sciences de la Vie et de la Sante, 33.

[B25] DRZE (Deutches Referenzzentrum fur Ethik in den Biowissenschaften) (1999). Ethik-Diskussion in der Gesellschaft durch Informationen unterstutzen.

[B26] Maynard A, Bloor K (1998). Our certain fate: Rationing in health care.

[B27] Richardson A, Charny M, Hammer-Lloyd S (1992). Public opinion and purchasing. British Medical Journal.

[B28] Abelson J, Forest PG, Eyles J, Smith P, Martin E, Gauvin FP (2003). Deliberations about deliberative methods: Issues in the design and evaluation of public participation processes. Social Science and Medicine.

[B29] Renn O, Weber T, Wiedelmann P (1995). Fairness and competence in citizen participation: Evaluating models for environmental discourse.

[B30] Neumann WR (1986). The paradox of mass politics: Knowledge and opinion in the American electorate.

[B31] Templeton F (1996). Alienation and political participation. Public Opinion Quarterly.

[B32] Arnstein S (1969). A ladder of citizen participation. Journal of the American Institute of Planners.

[B33] Litva A, Coast J, Donovan J, Eyles J, Shepherd M, Tacchi J, Abelson J, Morgan K (2002). The public is too subjective: Public involvement at different levels of health-care decision making. Social Science and Medicine.

[B34] Kathlene L, Martin JA (1991). Enhancing citizen participation: Panel designs, perspectives, and policy formation. Journal of Policy Analysis and Management.

[B35] Charles C, DeMaio S (1993). Lay participation in health care decision making: A conceptual framework. Journal of Health Politics, Policy and Law.

[B36] Splichal S (1999). Public opinion: Developments and controversies in the Twentieth Century.

[B37] Deutscher Bundestag (2002). Schlussberichit der Enquete-Kommission "Recht und Ethik der modernen Medizin" Drucksache 14/9020.

[B38] Abelson J, Forest PG, Eyles J, Smith P, Martin E, Gauvin FP (2002). Obtaining public input for health systems decision making: Past experiences and future prospects. Canadian Public Administration.

[B39] Einsiedel EF, Jelsoe E, Breck T (2001). Publics at the technology table: the consensus conference in Denmark, Canada, and Australia. Public Understanding of Science.

[B40] Guston DH (1999). Evaluating the first U.S. consensus conference: the impact of the citizens' panel on telecommunications and the future of democracy. Science, Technology, and Human Values.

[B41] Joly C (1999). Between consensus and citizens: Public participation in technology assessment in France. Science Studies.

[B42] Althaus SL (1998). Information effects in collective preferences. American Political Science Review.

[B43] McLeod J, Pan Z, Rucinski D, Glasser T, Salmon C (1995). Level of analysis in public opinion research. Public opinion and the communication of consent.

